# Can Implementing New Services Organization Models to Better Meet the Needs of Young People Bring About Practice Changes? Analysis of an Experiment in Québec

**DOI:** 10.1177/11786329241232299

**Published:** 2024-02-15

**Authors:** N. Touati, I. Ruelland, L. Rodriguez d’El Barrio, M. Bouchard, K. Beaulieu, I. Courcy

**Affiliations:** 1 Ecole Nationale d’Administration Publique, Montréal, QC, Canada; 2 Université Quebec a Montreal, Montréal, QC, Canada; 3 Université de Montréal, Montréal, QC, Canada; 4 Centre de recherche et de partage des savoirs InterActions, QC, Canada

**Keywords:** Youth, practice change, services organization, trajectories, health service integration

## Abstract

The research question addressed in this article is: Can implementing new services organization models to better meet the needs of young people bring about practice changes? More precisely, we examine the effects of a new model called Aire Ouverte (AO) which is implemented gradually across Quebec since 2019. This new model involves public sector and community organizations. To grasp practices’ change, we use cultural historical activity theory (CHAT) and employ a qualitative approach. Beyond a precise description of work activities, we gained an inside view of how the actors involved represented their practice and context. Our results show that practice changes seen by actors are in line with the object of the intervention, that is, responding rapidly to the expressed needs of young people. The development of new tools, flexible functioning, strengthening of interprofessional and intersectoral collaboration, involvement of young people in decision-making, all should contribute to improving response to their needs. This being said, a critical look at practice changes reveals a challenge in aligning the design and objective of AO with the needs of some young people. We noted also a poor alignment of effective collaborative practices between levels of care and the practices sought from intersectoral collaboration.

## Introduction

More than 10 years ago, scholars such Sauvignat^1^ drew attention to the amplification of social problems resulting from socio-economic crises and demographic trends. The public health crisis associated with COVID-19 exacerbated social problems such as homelessness, overdose deaths and mental health problems worldwide. Young people have been especially affected.^2-4^ Many analysts consider that these social problems, understood to be complex, demand closer attention to their multiple causes, stakeholders and territorial realities, and better management of the interdependencies between actors working in different sectors. For example, youth mental health issues concern actors from health and social services, education, employment, public safety, etc., none of whom have sufficient resources, legitimacy and expertise to meet the complex needs of young people. Interventions with vulnerable youth were historically piecemeal because services were designed to address specific problems. This specialization of services had the effect of fragmenting the person’s reality to fit with the mandate of each service organization and was detrimental to meeting their needs. To correct this situation, reform projects have sought to implement more holistic and territorial approaches to provide young people with a more coordinated response. Service networks have thus been deployed^5-7^ targeting vulnerable youth at risk of various forms of marginalization.^8^ Service integration is the process whereby professional and organizational actors deliberately coordinate their interdependencies using a variety of technical, informational, professional, and managerial strategies. This integration enables more consistent, efficient and better-quality interventions, while also improving the user’s experience of care and services.^9^

That said, more and more analysts now contend that meeting the needs of vulnerable population groups demands a paradigm shift that goes beyond better alignment of services to make the person’s needs the starting point for developing services.^10,11^ Notions of health and life trajectories have served to crystallize this paradigm shift.

*The trajectory perspective presupposes that each situation is unique, regardless of the broader category the pathology a person suffers from might fall under, because each person experiences the situation differently. Seeing thing in terms of trajectories supports a global view of all dimensions of a person’s life. It also emphasizes the importance of how people perceive their situation subjectively.*^10^ (p. 104).

This paradigm has shaped new service models that aim to improve response to young people’s needs: the Headspace model in Australia^12^; Foundry in British Columbia^13^; Jigsaw in Ireland,^14^ Access-Open Minds in Canada,^15^ Youth One Stop Shops in New Zealand^16^; Youth Wellness Hubs in Ontario,^17^ AireOuverte (AO) in Quebec.^18^ These models have been examined in many studies documenting their implementation^12,15^ and/or challenges in their implementation,^19^ the profiles of young people reached by the model,^14,15,20,21^ their impact on service utilization^22-24^; access barriers and facilitators,^25^ costs,^25^ and effects on young people’s health and/or social functioning^14,16,24^

A number of lessons can be drawn from the literature review, focusing on services integration for youth. By way of illustration, several studies suggest that locating several services in the same place, as is the case with the Youth One Stop Shops, Foundry and Headspace models, enables a better response to young people’s needs by facilitating access and engagement, avoiding the stigmatization of young people with mental health problems,^12,13,16,22,25^ and enhancing access to information on available services.^25^ Collaboration within multi-disciplinary teams also improves the informational continuity of services^12^ and their comprehensiveness. That said, collaboration with physicians can be a challenge.^25^ Collaboration with external partners from different sectors of public action and society promotes the diversification of services.^13^ However, the development of collaborative governance, including better power-sharing, requires a genuine cultural change.^26^ Finally, a number of studies^14,22,23,25^ have shown that the implementation of integrated service networks for young people leads to an increase in the use of mental health services, but that certain groups of young people are more difficult to reach. Studies on the effectiveness of these models are rather limited, as they usually focus on a single indicator such as psychological distress.^14,25^ Other methodological weaknesses also compromise the validity of these studies.^14,25^ In summary, it appears that these models of service organization are promising, but the realization of their potential depends on successful implementation, which translates into a transformation of practices.

To our knowledge, few studies have looked in depth at how the implementation of these models transforms professional and organizational practices. Some have looked at specific aspects such as interprofessional collaboration^27,28^ or practices that improve access to services.^15^ While interesting, these studies do not provide a global view that incorporates different dimensions of practice. A deeper analysis of practice transformations induced by the adoption of these new models is essential to grasp the magnitude of changes, their alignment with young people’s needs, and challenges around sustainability. Our study undertakes this analysis, looking at the AO model that has been implemented gradually across Quebec since 2019, and involves public sector and community organizations. The institutional context in Quebec makes the analysis especially interesting. Quebec has a long history of community action that has contributed significantly to social innovation.^29,30^ It also, in 2015, undertook a major reform resulting in the creation of megastructures—integrated health and social service centres (IHSSC)—designed, among other things, to facilitate access to services.^31^

The introduction of the AO model in Quebec was inspired by the experiences of other jurisdictions, in particular the Foundry (British Columbia) and Headspace (Australia) models. The Ministry of Health and Social Services initially decided to experiment with the AO model by funding 3 demonstration projects in 3 regions of Quebec, which ran from 2019 to 2023 and were accompanied by interdisciplinary research teams. The evaluation of this experimentation fed into the scaling-up of the model. From 2022, all integrated health and social service centers must implement the program. A reference framework has been drawn up to support the deployment of AO model.^18^ This framework formalizes the 5 guiding principles of the AO model, guiding important changes in practice in the field of youth intervention: (1) optimal accessibility and reduction of social health inequities, (2) for and with young people, (3) Co-designing partnerships, (4) flexible support and continuity, (5) holistic approach. By way of example, youth participation in decision-making is a new principle of governance in the Quebec context. By formalizing this principle, and adopting a top-down approach to change, the Ministry wished to force change and a departure from the status quo; while also demonstrating great leadership in accompanying change.

It is important to analyze how the introduction of the AO model in Quebec has given rise to effective changes in practices, in order to identify: (1) how actors in the field have appropriated this new model and (2) how it influences collaboration between the public and community sectors.

In so, the main research question addressed in this article is: what (professional, interprofessional and organizational) changes are produced by the implementation of the AO model, from the perspective of the practitioners and managers involved?

## Analytical Framework

We set out to empirically analyze professional practices, getting as close as possible to the actors involved.^32-35^ By carefully describing what actors do every day, it becomes possible to examine practices as they come into being at the intersection between formal and informal activities in organizational and inter-organizational contexts.^36,37^ It is important to consider that all forms of practice impose a “community of functions” between interdependent actors who participate for different reasons.^38,39^

**Figure 1.**
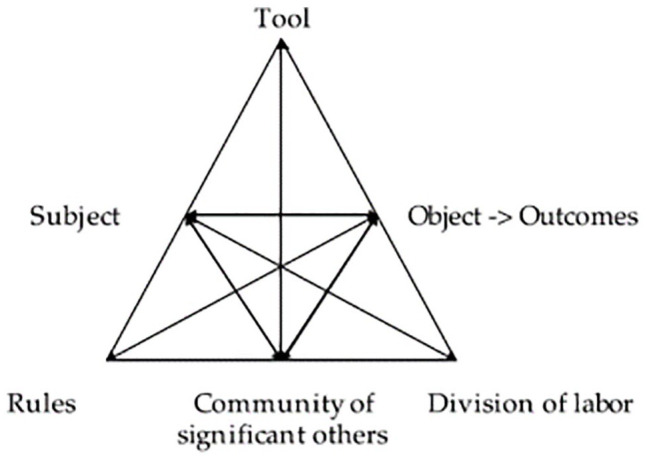
E 1 model of activity system. Source: Engeström (1987, p. 78).^34^

More specifically, we use cultural historical activity theory (CHAT), which provides a solid basis for analyzing work activities.^35^ This theoretical approach makes it possible to model the work activities of actors with different roles, positions and perspectives by locating them within an “activity system” composed of 6 main parts, each having cultural and historical dimensions. These are: (1) the **subject** (or protagonist of the action); (2) the **object** (the action’s target and/or expected results); (3) the **tools** used by the subject to act on the target or produce the desired results. Tools can be either material or conceptual. Therefore, protocols, methods and scientific models are considered tools on the same level as pencils, computers and telephones^1^; (4) the **community** involved in the activity, which refers to people who share the subject’s interest and involvement in the object. Finally, the relationship between subject and community is mediated by the last 2 parts of the activity system: (5) the **rules** that govern the subject’s actions toward the object and the relationship with other participants in the activity; and (6) the **division of labor**, understood as what is done by whom in relation to the object, including horizontal distribution of tasks and vertical distribution of power, positions, access to resources and rewards (Engeström, 1987, 1990 cited in Foot^35^). Foot^35^ represents these components as intersecting nodes in a triangular figure that represents an activity system (see Figure 1). Taking the plurality of interactions between these different dimensions into account enables us to understand work activities in a broader perspective by more fully integrating their real complexity.

This analytical framework makes it possible to draw on the expertise of all members of the research team from different disciplines, fostering a reflexive process across disciplines and with practice settings. By way of example, this framework crosses different dimensions of practice analysis, such as the role of rules, at the heart of the discipline of public administration, and the way subjects conceive and give meaning to their actions, at the heart of sociology and psychosociology. This trans-disciplinary approach has enabled us to gain a deeper understanding of practice change.

## Methods

This analysis of practice change is part of a larger participatory^40,41^ research project involving several components.^20,21^ The project seeks to transform practices through a process of co-constructing knowledge with researchers, young people, providers and managers and involved setting up an innovation lab.^42^ The research process respects all ethical requirements for research involving human subjects. An ethics certificate was issued by the IUHSSC of North of Montreal. For more details concerning the methods, see Annex 1.

We employed a qualitative approach to analyze practice changes.^43^ Qualitative methodology aims to “reveal the sense or meaning that the phenomenon under study holds for individuals”^43,44^ and enables research to answer how and why questions. More precisely, the analysis is based on a case study^45^ of the AO project implemented in Montreal, Quebec, as it is especially suited to understanding complex phenomena in context.

The 2 first authors conducted 39 one-on-one interviews with managers and staff working in the public health and social services sector and in community organizations serving the needs of young people in this part of Montreal. Interviews were semi-structured, as per Lutton’s^46^ definition, as interviews “[which] intended to purposefully pursue understanding (information and meaning), using predetermined questions, improvisational probes, and responsive follow-up questions.” Interviews addressed a number of themes: appreciation of the response to the needs of young people, understanding of the AO model, modifications brought to work routines, challenges in practice change, etc. For more details, see interview grid (Annex 2). The design of the interview grid was inspired by our theoretical framework. The first interviews allowed to test the clarity of the questions.

In addition, in-situ observation (200 hours) of meetings (bringing together professionals and managers from AO) and innovation labs (also engaging stakeholders from the community sector), involving all the team members, was carried out. The information provided enabled a better understanding of the dynamics and issues facing the actors. The observations and participatory structure of the research allowed a contextualized analysis of the data collected in interviews.

All participants were informed of the conditions of their participation and freely provided their consent. Interviews were recorded and transcribed verbatim.

Data were assembled and coded (see coding grid in Annex 3) using QDAMiner software (version 4), and the process was validated through constant exchanges between team members. Finally, the collected and triangulated data were analyzed thematically^47^ based on our analytical framework.

## Results

### Object

Data analysis revealed an important change in the way AO team members and managers saw the purpose of their action. Their priority became responding rapidly to the expressed needs of young people. This meant putting greater emphasis on their lived experience, independent of institutional norms (eg, diagnosis). It also meant that, in contrast to other programs where the distinction between evaluation and service generated wait lists, immediate action was the priority.

*I see a particular openness in AO practitioners; it may be there in some practitioners in other programs, but it’s generalized in AO where there are no wrong questions, wrong needs, wrong times or wrong places; we take them where they are, as they are and if they don’t ask for anything, we welcome them anyway. If they knock on the door or call or text it’s because they have a need, even if it isn’t clearly defined at first; we’ll talk, go for coffee, it’s a very different approach that’s centred on where the young person is when we meet them and to me that characterizes AO (Manager)*

Workers consider that AO’s objective is to offer a safety net for young people who have trouble getting their needs met adequately, while waiting for other organizations or programs to take over.

*It requires a way of thinking about limits; you may find this strange, but as soon as the youth arrives here and we connect with them, we have to think about OK, where are we going to send them, what is it they need beyond us, so that we don’t become another mental health program or another. . . we’re a sort of roundabout at AO, where there are opportunities to exit; you may go around a few times but at some point you have to head off down the road towards somewhere else.(Practitioner)*

That said, they consider that the AO team must help the young persons find their way in the system.

*There’s one word that defines AO, and that’s accompaniment. We will accompany you in our network as a kind of system GPS. Young people are disoriented, they don’t know our services. I can tell you what you can do, what you can’t do, your rights and your responsibilities, and I’ll accompany you because I know it’s hard. (Practitioner )*

### Subjects

The AO team, which plays a major role in the model, includes various professionals, specialized educators, social workers nurses, sex therapists, and community organizers.^2^

Along with understanding the objective of their own practice, these subjects see themselves above all as members of a team and, in this way, put the emphasis on their shared competencies in community based psychosocial intervention.

*There’s the whole issue of reaching out as well that calls for different kinds of skill; there’s a big difference between getting a request for services from someone who comes in on their own, and having to put things in place to go. . . you know, it may be a young person who’s all alone at home and never goes out anymore and doesn’t want services, so how do I go about reaching out to them? That’s the kind of problem we talk about and makes us evolve as professionals (Practitioner )*

In other words, the professional identity of workers is built on common skills imposed by this community work rather than a particular discipline. This psychosocial orientation focused on skills in participatory and interprofessional work can sometimes develop at the expense of specific professional skills. Some workers feel less equipped to respond to specific problems, such as blood-borne and sexually transmitted infections (STIs). Professionals such as nurses sometimes feel alone in their work and lack the opportunities to confirm their clinical decisions with people who have the same expertise.

### Tools

Consistent with changes in how AO workers and managers see the object of interventions, changes are also seen in the tools they employ.

Physical spaces were redesigned to make young people more comfortable and increase the likelihood of their coming to find AO services. It was important that AO spaces not look like institutional structures. Workers described the significant effort put into designing spaces, in collaboration with young people (cf. community). A mobile unit was also designed to enable AO teams to better reach young people. To this end, workers also had permission from their employer to make greater use of technological tools, and especially email and text messages. This option, which is not available in other programs, brought considerable added value. Use of information technology comes up against strong institutional constraints (eg, prohibition to use Instagram).

*Our phones facilitate access, regardless of the means the youth use to contact us; we get a lot of emails. They’re technological beings and even those who are hard up have cell phones. It’s how they communicate. Workers in other youth programs didn’t have that tool before. But with AO we have to adapt our practice to really create a connection with youth and hook them into the network to support them. Email is a wonderful tool, it’s less confrontational. If they can say, I’d like to see a nurse, could I make an appointment, that’s less intimidating for a young person with generalized anxiety or social phobia or whatever. (Practitioner )*

AO team members mentioned several other tools that helped strengthen teamwork (cf. division of labor). The design of workspaces, and especially the fact that all shared the same space, was helpful as it made communication more fluid and spontaneous. The creation of a visual station to follow cases improved coordination. As well, a new system for taking notes allowed all team members to stay informed about young people’s evolving needs and the interventions each of them undertook to meet these needs.

*From the start all eight of us were in the same office and we shared everything. The work really felt collegial and multidisciplinary. (Practitioner )*

### Division of labor

Team members insisted that meeting young people’s needs requires greater interprofessional collaboration, which was already well established in their practices. However, in AO, this collaboration also extended to an intersectoral level (cf. community). As well, in AO collaboration could involve actual co-interventions, a change that was regarded as highly significant.

*In fact, co-intervention is much simpler, more legitimate and acceptable in AO than in other programs where there are long wait lists and we can’t afford to put two workers on the same case. (Manager)*

As well, interprofessional collaboration within the AO team, and more specifically between psychosocial providers, brought greater flexibility in sharing cases, and this in a way consistent with each worker’s professional identity (cf. subject). Cases were assigned according to the worker’s comfort in dealing with the young person’s problem.

*A young person who arrives at AO is not immediately labelled as belonging to a particular practitioner. They come to AO to see someone, so we’re all on equal footing, whether an educator, social worker, we’re there to listen to them. So, the young person is coming to AO and not to the social worker (name). I find that more inclusive, less administrative and more community-minded; it’s good. We ask who is comfortable with this case, who wants to take it, and decide from there. (Practitioner )*

Beyond taking on individual cases, transversal responsibilities (eg, being on call, developing partnerships) are shared among team members. While the collegial way of functioning is seen in a positive light, some team members also recognize potential adverse effects.

*What’s good about AO is that we share all duties. But at the same time, it can be, how should I put it. You know when a duty is shared, no one person is responsible for it, we’re all involved, you know what I mean? But sometimes when everyone does something, then nobody does it. (Practitioner )*

### Rules

The AO project involves significant changes to the rules that govern both the interventions of professionals in the team and collaboration between the team and different partners on the territory.

As previously mentioned, 5 principles^18^ determined by the Ministry of Health and Social Services are at the heart of the AO model and steer the team’s practices. AO practitioners wholeheartedly embrace them, as do most partners within the IHSSC, schools and the community sector (notably among organizations focused on employability). These principles are seen as an innovative reference point with considerable potential to meet young people’s needs differently and better. The AO team’s flexible mode of operation is regarded as a key asset. This flexibility translates into openness and inclusion (young people do not have to live on the territory to get help, they can choose their intervener and the place and time of the intervention (extended schedule) and margins of freedom are cocreated to adjust to emergencies (as when one youth brought all their belongings to the AO office while waiting for housing, or cases that remain open for a long time). The team tends to take on almost all needs in order to then refer people to the appropriate resources. However, the organizational conditions that enable this flexibility are fragile and, according to some, ephemeral:

*I’ve seen it in other programs that were seen as the best of the best and benefitted from a whole lot of money but also left accountability a little loose. (. . .) In terms of performance and other expectations. So it gave teams a chance to do things differently, to dedicate themselves entirely, sometimes have lighter caseloads, fewer performance requirements. . . And what I’ve seen in the past is that when the novelty wears off, it changes. (Manager)*

Community organizations on the territory working with youth recognize this flexibility as relevant and in line with their mission and mode of intervention. However, some fail to see the added value, as they already work with alternative approaches based on the needs and participation of young people.

### Community

A network of services is engaged in better meeting young people’s needs (cf. object), including community organizations, other IHSSC programs (youth, mental health), public organizations (schools, municipalities) as well as young people and their families in Montreal North.

Within the IHSSC, the services of the AO team are regarded as a useful resource for youth aged 12 to 25 years who are farther away from conventional services. The fact that AO is a ministerial initiative supported by IHSSC leadership has not, however, overcome access difficulties to services such as pediatric psychiatry or family medicine. Despite AOs having signed agreements with medical clinics to facilitate access, demand exceeds supply. This breeds community organization dissatisfaction with AO’s contribution to meeting young people’s needs.

*We had hoped that that was something AO could do, because we can’t. AO is inside the network. Do things like establish a diagnosis and find a psychologist or psychiatrist to begin therapy; we can’t do that, and we hoped that the young person could go to AO and be connected to long-term follow-up. We would like to refer people to AO and say: this young person seems to have major mental health problems and is not being followed and must get his drug use under control, for example. We can help to a certain extent but we’re not psychotherapists. So can you, can they go see you, for workshops or group sessions on drug use to help them take control before it’s out of control. But that’s not what we see at the moment. (Community worker)*

Few community organizations actually refer the young people they see to AO. Some feel that they already provide the services available from the AO team. Others do not see the usefulness of collaborating with AO other than when a youth is in acute crisis.

*Honestly, we were just asking ourselves the question today, about their role and responsibilities. At one point, we thought all they did were referrals; later we found that my God no, they’re actually providing services, and then, from our perspective, we see hey they’re starting a mobile unit, that’s what community organizations do, how is it they started doing that? That’s the kind of question we have; I wasn’t expecting a CIUSSS to become one of us, I wasn’t expecting that, that’s what we’re here for; I was expecting something different. (Community worker)*

A number of strategies were used by the AO team to advertise AO and promote intersectoral collaboration, including information sessions, participating in youth tables and holding co-construction sessions in the innovation lab with community actors and youth. These strategies helped others understand AO’s role and improved collaboration with the community. For example, community organizations were consulted frequently when developing the mobile unit project. Over time, schools and community organizations offering work integration programs called on the team more frequently for mental health supports or sexually transmitted infection screening. Real interprofessional and intersectoral collaboration was also achieved to respond rapidly to young people facing complex problems (eg, a social worker from the AO team, a school-based worker and a community shelter resource to act on a situation of domestic violence one youth experienced).

To strengthen collaboration, service agreements were signed with 6 community organizations and 3 youth tables to establish co-development structures and create AO service outlets within community partner and school settings. Joint initiatives were created, notably in mental health.

Finally, strategies were developed to meet ministerial requirements for participation by young people and their families, with the establishment of a youth committee.

They listened to what young people wanted, recognizing the limits to what is possible in the AO team’s institutional setting. For example, some requests around means of communication were refused. The team members’ flexibility and ingenuity often found ways to work around limitations, while innovating in terms of participation. Examples include one team member’s initiative to co-construct a playlist for background music in the AO space. Community organizations saw such developments positively, despite some feeling it duplicated their own approaches.

## Discussion

Analysis of practice changes seen by members of the AO team reveals that many changes, including some also seen in the implementation of the ACCESS Open Minds model,^15^ are in line with the object of the intervention. The development of new tools, flexible functioning, strengthening of interprofessional and intersectoral collaboration, involvement of young people in decision-making, all should contribute to improving response to their needs.

We find that these changes are extensive, at least in the Quebec context. Implementation of the AO model creates action spaces that are antithetical to how health and social service organizations usually function in Quebec, which generally focus on control and accountability,^48^ and thereby reduce the power of professional staff. Based on analyses of the adverse effects of such mechanistic models, Dupuis and Farinas^48^ advocate the development of practices that more closely resemble organic organizations, with an emphasis on flexibility.^49,50^ This is the aim of the AO model, which seeks practice changes that consider the complexity of young people’s needs. That said, the effectiveness of the model rests on the overall coherence of practice changes. A number of empirical studies have shown that the effects of complex interventions depend on the alignment between these characteristics and the action context.^51^ However, a critical look at practice changes seen in this study reveals a challenge in aligning the design and objective of AO with the needs of some young people. Taking on the role of rapidly finding place for a young person in another health and social service resource (cf. object) can work against meeting their needs, knowing that they will have trouble establishing trust relationships with these new providers.

There is also poor alignment of effective collaborative practices between levels of care and the practices sought from intersectoral collaboration. As seen in results, the failure to solve problems with access to pediatric psychiatry and primary care impede collaboration between the AO team and community organizations. It is difficult within a pilot project to address these issues; they affect the entire population and public authorities have yet to find satisfactory solutions, at least in the Quebec context, partly due to doctors’ independent status that complicates the regulation of medical practice. Other aspects of the institutional context in Quebec influence the collaborative dynamic between the AO team and community organizations. Community organizations have long made significant contributions to Quebec society, notably in social innovation, and consider that these are insufficiently recognized in funding formulas. In this context, they take a poor view of the public sector imitating the best practices of the community sector rather than providing it with adequate financial support.

Despite these difficulties, analysis of the implementation of the AO model demonstrates significant advances in terms of practice change, notably around rules of operation, and youth participation in governance. The adoption of a top-down change management approach has effectively enabled actors to break out of the status quo and foster the emergence of new practices, thanks to a learning process encouraged by experimentation.^52^ This begs the question of how long these changes are likely to last. Results from our study suggest that advances are extremely fragile. In the absence of strong incentives for collaboration, especially in terms of visibility and reputation incentives^53^ that are very present during the experimental phase, the AO team may lose the conditions that enable innovation. If, as well, pressure from demand for services increases, the organization may lose its room to maneuver in terms of resources (organizational slack)^54^ that enables it to explore new ways of doing things. The risk of reverting to the status quo is real. As Grenier and Denis^55^ (p. 200) remind us: “(. . .) innovation often collides with forces of reproduction, especially the control held by elites over policy directions and strategies, and the weight of existing institutions.”

In our view, this study makes two contributions to the state of knowledge on the adoption of new integrated service models for young people. Firstly, our multi-dimensional analysis of practices highlights the need for coherence between the different dimensions of practice, which is essential if they are to be effective in meeting the needs of young people (cf. alignment issues). Other studies that have analyzed practice change have focused on certain aspects, for example collaboration between team members^26,27^ and therefore do not examine, or at least not as explicitly and deeply, how different dimensions interact with each other. Such an approach does not allow us to see how, for example, the degree of collaboration between professionals attached to different levels of care influences collaboration with community organizations. Our understanding of these issues of coherence has been enriched by cross-referencing the views of public and community actors on changes in practice.

Our second contribution relates to the tensions that characterize inter-sector collaboration, between public and community organizations around the needs of young people. These tensions are rooted in a particular institutional context (cf. Introduction) and reflect, among other things, issues of recognition of community action. A number of studies have already documented the challenges of collaboration between the public sector and community organizations, particularly with regard to the needs of young people^56^ and more generally.^57^ In particular, these studies have highlighted the harmful effects of competition, accountability and the imposition of a government agenda on collaboration between the state and the third sector.^56,57^ Beyond these issues, this study reveals other sources of tension, stemming from a perception by community organizations of a lack of recognition of their expertise, resulting in imitation of their good practices by the public sector. This recognition is seen as weak, in that it does not translate into a fair sharing of resources and powers over the organization of services.^58^ To reduce these tensions, there is a need to work toward greater symbolic and material recognition of the contribution made by community organizations, and to build consensus on priorities for meeting the needs of young people.

In conclusion, thanks to a qualitative research methodology, our study sheds light on the changes in practices resulting from the adoption of new service organization models aimed to better meeting the needs of young people, based on a new paradigm centered on the notion of health and care pathways. The lessons learned from this study may be useful for decision-makers and researchers interested in issues of collective action around the complex needs of so-called vulnerable populations.

In future research, we will explore how the practices of AO teams continue to evolve in a context of radical change in the socio-sanitary network, and how the maintenance of innovation laboratories, nurtured in particular by the participation of young people, contributes to the renewal of practices. Finally, we’ll be looking at how these new practices improve the response to young people’s needs.

The limitations of the study must also be mentioned. As with any qualitative study, we cannot claim to be able to generalize. On this point, it would be interesting to carry out a comparative analysis of different AO demonstration projects set up in Quebec to see how collaboration and territorial characteristics can generate different experiences in terms of changes in practices. In addition, the point of view of youth and their families would be a valuable addition to our analysis of changes in practices.

## Supplemental Material

sj-docx-1-his-10.1177_11786329241232299 – Supplemental material for Can Implementing New Services Organization Models to Better Meet the Needs of Young People Bring About Practice Changes? Analysis of an Experiment in Québec Click here for additional data file. Supplemental material, sj-docx-1-his-10.1177_11786329241232299 for Can Implementing New Services Organization Models to Better Meet the Needs of Young People Bring About Practice Changes? Analysis of an Experiment in Québec by N. Touati, I. Ruelland, L. Rodriguez d’El Barrio, M. Bouchard, K. Beaulieu and I. Courcy in Health Services Insights

sj-docx-2-his-10.1177_11786329241232299 – Supplemental material for Can Implementing New Services Organization Models to Better Meet the Needs of Young People Bring About Practice Changes? Analysis of an Experiment in Québec Click here for additional data file. Supplemental material, sj-docx-2-his-10.1177_11786329241232299 for Can Implementing New Services Organization Models to Better Meet the Needs of Young People Bring About Practice Changes? Analysis of an Experiment in Québec by N. Touati, I. Ruelland, L. Rodriguez d’El Barrio, M. Bouchard, K. Beaulieu and I. Courcy in Health Services Insights

## Notes

1. Foot^35^ specifies that “*tools are crafted at a point in time and adapted over time– their development is shaped by the needs, values, and norms of the culture(s) in which they are created and used*” (p. 5).

2. Not initially identified as team members in the model.
