# Supplementary material for: Can Implementing New Services Organization Models to Better Meet the Needs of Young People Bring About Practice Changes? Analysis of an Experiment in Québec
Source: Health Serv Insights. 2024 Feb 15;17:11786329241232299. doi: 10.1177/11786329241232299 (PMC10874162; doi:10.1177/11786329241232299)
Supplement: sj-docx-1-his-10.1177_11786329241232299 – Supplemental material for Can Implementing New Services Organization Models to Better Meet the Needs of Young People Bring About Practice Changes? Analysis of an Experiment in Québec [file sj-docx-1-his-10.1177_11786329241232299.docx]

**North of the Island of Montreal Aire Ouverte Demonstration Project**

**Research – Process section**

**Coding tree**

**Meta category // respondent characteristics**

**ROLE:**

- Aire ouverte (AO) manager
- Manager of another program within the Integrated University Health and Social Services Centre (IUHSSC)
- AO team professional
- IUHSSC professional partner
- Education sector partner
- Community sector partner

**1. Youths and services (before and beyond After AO implementation)**

1.1 Youths reached (characteristics, group affiliations, needs, challenges)

1.2 Description of means used to meet youths’ needs

1.3 Facilitating factors (how services reach youths, new ideas and means, descriptions)

1.4 Hindering factors (criticism, systemic issues, difficulties in reaching youths)

**2. Collaboration to meet youths’ needs (before and beyond AO)**

2.1 Collaborative experiences to meet youths’ needs

2.1.1 Actors (types of stakeholders, organizations, youths)

2.1.2 Collaboration objects

2.1.3 Collaborative actions

2.2 Appreciation of the collaboration to meet youths’ needs

2.2.1 Qualification (good, bad)

2.2.2 Facilitating factors

2.2.3 Hindering factors

2.2.4 Results

2.2.5 Courses of action for future collaboration

**3. The AO model**

3.1 Provenance of knowledge of AO

3.2 Comprehension and appreciation of the AO model (Is it innovative? Is it a good solution?)

3.3 Description of youths targeted by AO

**4. Implementation process**

4.1 Implementation actions (choosing a site, adapting the schedule to youths, recruiting employees, presenting AO to the IUHSSC board of directors, presenting AO to various community stakeholders, hiring sexologists, acquiring a recreational vehicle, etc.)

4.2 Governance spaces

4.2.1 Innovation spaces

4.2.2 Administrative spaces (ex: IUHSSC level monitoring committee, ministry level committee, etc.)

4.2.3 Concertation (ex: with community organizations when not working on changing practices, with neighborhood and youth round tables)

4.2.4 AO youth advisory committee

4.2.5 Other spaces, informal, etc. (meetings)

4.3 Implementation appraisal

4.3.1 General appreciation

4.3.2 Facilitating factors

4.3.3 Hindering factors

4.3.4 Recommendations

**5. Practice change**

5.1 Youths reached (characteristics, group affiliations, needs, challenges, youths reached or not)

5.2 Professional practices (roles, description of work activities, how practices unfold, professional identity)

5.2.1 Description

5.2.2 Facilitating factors

5.2.3 Hindering factors

5.3 Interprofessional practices

5.3.1 Actors

5.3.2 Collaboration objects

5.3.3 Coordination mechanisms (ex: team meetings, shared files, etc.)

5.3.4 Tensions and resolution strategies

5.3.5 Facilitating factors

5.3.6 Hindering factors

5.4 Appreciation of collaborative practices within AO and with AO

5.4.1 Results (ex: improved referral, improved relations between the health and social services network and the community sector, improved access to services for youths)

5.4.2 Considerations for the future of AO (challenges and recommendations)

**6. Emerging themes**

6.1 COVID-19

6.2 Other
