# Supplementary material for: Can Implementing New Services Organization Models to Better Meet the Needs of Young People Bring About Practice Changes? Analysis of an Experiment in Québec
Source: Health Serv Insights. 2024 Feb 15;17:11786329241232299. doi: 10.1177/11786329241232299 (PMC10874162; doi:10.1177/11786329241232299)
Supplement: sj-docx-2-his-10.1177_11786329241232299 – Supplemental material for Can Implementing New Services Organization Models to Better Meet the Needs of Young People Bring About Practice Changes? Analysis of an Experiment in Québec [file sj-docx-2-his-10.1177_11786329241232299.docx]

| 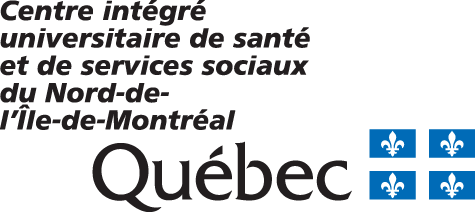 | 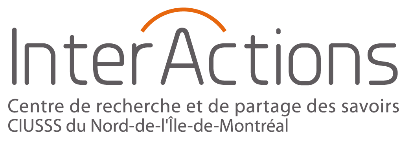 |  |
| --- | --- | --- |

**AIRE OUVERTE MODEL: INTERVIEW GUIDE**

**PART 1: BACKGROUND**

**Objective:** situate the positions of the actors involved in the implementation of the Aire ouverte model and their context of action

**A) The profile of the respondent**

Sub-objective: know the respondent and their position in the action system.

Can you give us an overview of your experience with young clients ?

- What is your field of study?
- How long have you been working with “young” clients?
- Can you explain to us generally your role in the implementation and appropriation of Aire ouverte practices? (Note: theme which will be explored further below)
- What motivated you to join the Aire ouverte project? How long have you been involved in this project?

**PART II: THE AIRE OUVERTE MODEL**

**Objective:** understanding the different conceptions of respondents in relation to the Aire ouverte model as well as their analyzes of the processes and practices implemented to meet the needs of young people

**B) Understanding the Aire ouverte model**

Sub-objective: understand the respondent’s conception of the Aire ouverte model.

These questions relate specifically to the Aire ouverte model

- How would you describe the Aire ouverte model in a few sentences?
- What is the profile of young people targeted by Aire ouverte?
- To what extent do you think the model is a good solution to meet the needs of young people?
- Do you consider this model to be “innovative”? If yes, explain how. If not, can you justify your answer?
- What elements of the model do you think require further clarification?

**C) The population served**

Sub-objective: understand the respondent's perception regarding the issues and needs of young people targeted by Aire ouverte and the responses to these needs.

Now let's talk about the youth clientele that you reach.

- Can you describe the specific needs of this clientele? How and to what extent do you succeed in meeting their needs?
- Which young people are more difficult to reach by Aire ouverte? What efforts could be made to reach them in the future and better meet their needs?
- What are the factors that facilitate the response to the needs of young people through Aire ouverte? Conversely, what are the factors that make it difficult to meet the needs of young people?

**D) A look at the Aire ouverte implementation process**

Sub-objective: understand the critical analysis of the respondent in relation to the implementation process

- What is your general assessment of the measures adopted (integration measures, training offered, clarification of roles and coordination mechanisms, etc.) to implement AO before your involvement in the project?
- What has been done since you joined the project? What do you think are the most structuring actions to implement AO?
- What is planned in the short term?
- What overall assessment do you make of these first stages of the implementation of Aire ouverte?

**E) Look at the governance (coordination modalities) of AO**

Sub-objective: understand the respondent’s critical analysis of governance and its spaces

We will discuss here the spaces (internal and external), which allow a connection between the actors concerned by AO, with a view to coordinating the work together.

- Can you name some spaces that participate in the coordination in which you participate?
- In what discussion/decision-making spaces have you been involved? (For example: Discussion table, innovation laboratory, implementation team; coordination team, etc.)
- What is your assessment of the “functioning” of the different spaces in which you are involved (for example, climate, sharing of speaking, sharing of decision-making power, relevance of the subjects of discussion, management of tensions)?
- In which spaces did you feel particularly at home and felt that you contributed “to making a difference” (alone or with others)? and why ?
- Which spaces work best from your point of view? More precisely, what are the spaces which, according to you, have allowed an enrichment of the understanding of the problems and the reflection on the changes underway?
- What is your assessment of the place given to young people in these spaces? What were the effects of this involvement?
- To what extent does Maison Sauriol as a physical space influence internal and external coordination with the actors of the AO network? (the fact of having a location, its characteristics, the possibility of receiving partners, schedules, etc.).

**F) Insights into changes in professional (and inter) and organizational (intra- and inter-organizational) practices**

Sub-objective: Capture the critical analysis of the respondent on the practices implemented to meet the needs of young people and their transformation.

Here, we are interested in the changes in practices observed at your level (professional, inter-professional and inter-organizational practices) which are deployed to meet the needs of young people.

***F.1. Changes in professional practices and within the team (inter-professional)***

- To what extent Aire Ouverte has brought about changes in professional practices and in interprofessional collaboration relationships.
- Can you describe these changes in practices?
- What changes do you think are the most promising and, conversely, the least promising, from the point of view of responding to the needs of young people? Can you give us examples?
- In your opinion, what are the factors that facilitate or, on the contrary, hinder the development of your professional practices?

N.B: if the issues of the pandemic do not emerge, ask the following question: To what extent have you had to adapt your practices in the context of a pandemic? Can you give us examples? To what extent do you think these practices will continue beyond the pandemic context?

***F.2. Changes in service usage***

Hierarchy of services (proximity services, 1st, 2nd and 3rd lines of the SSS network)

- To what extent Aire Ouverte has promoted greater use of front-line services (proximity) and less use of specialized services (e.g. emergency services, youth center, hospital services, etc.)
- Have you noticed any differences depending on the territories and situations of young people and families? If so, can you illustrate?
- In your opinion, what are the factors that promote or harm these transformations?

***F.3. Collaborative practices around the needs of young people and families***

Here we will talk more specifically about young people and families who experience complex situations (several complex needs)

- Can you describe examples of collaborative practices (internal and/or external… intersectoral) to respond to complex situations experienced by young people and families? Any positive and/or critical experiences? Can you give us examples related to issues (sectors) of academic success, employability, mental health, immigration, etc.?
- Have you noticed any differences depending on the territories and/or situations of families and young people?

N.B: if the issues of the pandemic do not emerge, ask the following question: To what extent have you had to adapt your collaboration practices in the context of a pandemic? Can you give us examples? To what extent do you think these practices will continue beyond the pandemic context?

***F.4. Initiatives to improve collaboration***

- With which actors are you working to develop internal and external collaboration to meet the needs of young people? Can you describe these initiatives and their impact? What are the factors and contexts that favor or harm these approaches?

***F.5. Assessment of changes in practices***

- What, in your opinion, are the main challenges for the evolution of the Aire Ouverte clinical project?
- If you had to make a recommendation regarding the implementation of the Aire Ouverte (AO) model elsewhere in other territories, what would it be? Scaling up: advice for implementing AO in other districts at the CIUSSS or in other areas or territories?
- Would you like to add anything?

Thank you for your collaboration! If you wish, we could ask you for a second individual interview and for participation in a meeting to discuss the results of our preliminary analyzes.
